# Potential Role of Plasmalogens in the Modulation of Biomembrane Morphology

**DOI:** 10.3389/fcell.2021.673917

**Published:** 2021-07-21

**Authors:** Zakaria A. Almsherqi

**Affiliations:** Department of Physiology, Yong Loo Lin School of Medicine, National University of Singapore, Singapore, Singapore

**Keywords:** plasmalogen, biomembranes, dynamic system, cubic membranes, membrane morphological changes

## Abstract

Plasmalogens are a subclass of cell membrane glycerophospholipids that typically include vinyl- ether bond at the sn-1 position and polyunsaturated fatty acid at the sn-2 position. They are highly abundant in the neuronal, immune, and cardiovascular cell membranes. Despite the abundance of plasmalogens in a plethora of cells, tissues, and organs, the role of plasmalogens remains unclear. Plasmalogens are required for the proper function of integral membrane proteins, lipid rafts, cell signaling, and differentiation. More importantly, plasmalogens play a crucial role in the cell as an endogenous antioxidant that protects the cell membrane components such as phospholipids, unsaturated fatty acids, and lipoproteins from oxidative stress. The incorporation of vinyl-ether linked with alkyl chains in phospholipids alter the physicochemical properties (e.g., the hydrophilicity of the headgroup), packing density, and conformational order of the phospholipids within the biomembranes. Thus, plasmalogens play a significant role in determining the physical and chemical properties of the biomembrane such as its fluidity, thickness, and lateral pressure of the biomembrane. Insights on the important structural and functional properties of plasmalogens may help us to understand the molecular mechanism of membrane transformation, vesicle formation, and vesicular fusion, especially at the synaptic vesicles where plasmalogens are rich and essential for neuronal function. Although many aspects of plasmalogen phospholipid involvement in membrane transformation identified through *in vitro* experiments and membrane mimic systems, remain to be confirmed *in vivo*, the compiled data show many intriguing properties of vinyl-ether bonded lipids that may play a significant role in the structural and morphological changes of the biomembranes. In this review, we present the current limited knowledge of the emerging potential role of plasmalogens as a modulator of the biomembrane morphology.

## Introduction

Biomembranes are of fundamental importance in providing cellular compartmentalization and regulation of intracellular activities. An extensive review of the literature reveals that biomembranes are dynamic as they can transform into various morphological architectures and shapes. Biomembrane transformation could be a selective process to fulfill a specific purpose under the continuous and ever-changing intracellular and extracellular environment.

 The physical and chemical characteristics of the biomimetic materials determine the structure and morphology of the artificial membranes (Jurak et al., 2018). Similarly, the physicochemical properties of the biological membranes are determined by the membrane’s proteins and phospholipids composition. Their unique combination influence the geometry, morphology, and function of the biomembrane. Morphological changes of the biomembrane are often related to the overexpression of certain membrane-resident proteins and, to a lesser extent, to the changes in the membrane lipid profile (Almsherqi et al., 2009). The role of membrane lipids in this regard was speculated based on published reports of biomembrane transformation in mammalian cells upon overexpression or inhibition of key regulatory enzymes of the lipid and cholesterol synthesis such as HMG-CoA reductase (Almsherqi et al., 2009).

In principle, any amphiphilic molecule is capable of inducing membrane transformation. Based on the chemical structure of the lipid bilayer and its interactions with the membrane proteins, several membrane patterns could be generated. Evidence from *in vitro* studies clearly shows that the propensity of membrane-forming lipids to initiate non-lamellar structures is pivoted mainly on their molecular shape concept (Epand, 1997, 2007). For example, biomembranes may adopt different morphologies such as plain lamellar, stacked lamellar, whorls, or hexagonal depending on the phospholipid headgroup size and charge (Epand, 1997; Madrid and Horswell, 2013). Further, the changes in shape and structure of biomembranes are also influenced by the alkyl chain length (e.g., very long chain fatty acids, VLCFA) and the number of double bonds of the unsaturated phospholipid (polyunsaturated fatty acids, PUFA; Deng et al., 2009). More specifically, the alkyl chain length and the degree of unsaturation of the phospholipid are foundational to the structural impact on the complex lipid derivatives in biomembranes. Phospholipids with highly unsaturated docosahexaenoic acid (DHA) or docosapentaenoic acid (DPA) alkyl chains are usually found in cholesterol depleted, non-raft membrane domains (Wassall and Stillwell, 2008), while lipid rafts domain are enriched in arachidonic acid (Pike et al., 2002). The introduction of DHA-rich domains is speculated to induce changes in the conformation of signaling proteins (Wassall and Stillwell, 2008) and to induce non-lamellar, highly curved membrane structures such as hexosomes in DHA-monoglyceride mixture (Yaghmur et al., 2019) or hexagonal and cubic membrane organizations (Deng et al., 2009). Interestingly, the structural impact of omega-3 and omega-6 PUFAs, for example, are profoundly different, in terms of their influence on biomembrane morphology, although the only difference between the two molecules is their respective positions of the double bonds on the acyl chain (Deng et al., 2009).

**FIGURE 1:**
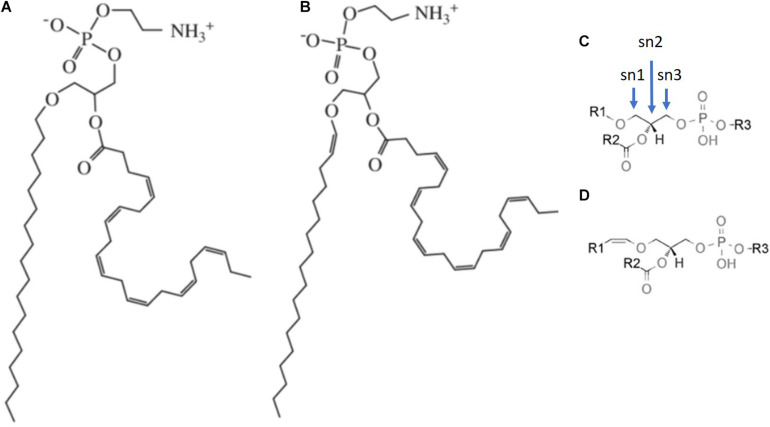
Structure of ether-linked membrane lipids differing in the sn1 linkage. **(A)** Alkyl chain linked to sn1 position via an ether-linkage (plasmanyl ethanolamine) and **(B)** Alkyl group linked to sn1 position via a vinyl ether linkage (plasmenyl ethanolamine). The general structure of **(C)** plasmanyl and **(D)** plasmenyl phospholipid species.

Besides studying the roles of VLCFA, PUFA, and cholesterol in inducing lipid membrane structural changes, recently, there is a heightened interest in investigating the role of ether phospholipids in biomembrane morphology. Ether phospholipids are ubiquitous and occasionally they are the major components of the cell membranes of anaerobic bacteria, primitive protozoa, fungi, and in the mammals’ cell membranes largely in the nervous, cardiovascular and immune systems (Braverman and Moser, 2012; Jiménez-Rojo and Riezman, 2019). There are two types of ether phospholipids, namely, plasmanyl phospholipid, and plasmenyl phospholipid (also known as plasmalogen). Typically, the sn-1 positions in plasmanyl and plasmenyl lipids are occupied by either 16 or 18 carbon attached via ether or vinyl-ether moieties, respectively, (Figure 1). In plasmalogens, the sn-2 position often carries PUFAs (Braverman and Moser, 2012; Dorninger et al., 2015). Indeed, ester, ether, and vinyl-ether lipids have their unique structures and physicochemical characteristics that affect biomrmbranes morphology, related to the cross-sectional area of the lipids, the differences in packing, and the interaction with other membrane components such as proteins and cholesterol (Casares et al., 2019). There is an increasing body of evidence demonstrating that vinyl-ether phospholipids have a direct effect on the membrane physicochemical properties including the rigidity/fluidity, fission/fusion, and morphological transition, (Koivuniemi, 2017) while ether lipids have been linked to complex cellular dysfunctions manifested as neurodegenerative and neurodevelopmental disorders (Dorninger et al., 2017). In this review, we mainly focus on plasmalogens, since they represent the major constituents of ether lipids in mammalian membranes, and because the biomembrane structural and morphological changes associated with impaired ether lipids are usually linked to the alteration of plasmalogen levels.

Plasmalogens have a multistage evolutionary history and a unique distribution across living organisms (Goldfine, 2010). Approximately 1 in 5 phospholipids are plasmalogens in human tissue where they are especially enriched in the neuronal, cardiac, and immune cells (Braverman and Moser, 2012). For example, choline plasmalogens account for around 30–40% of choline glycerophospholipids in human heart tissues, whereas ethanolamine plasmalogens account for around 70% of ethanolamine glycerophospholipids in myelin sheaths (Farooqui and Horrocks, 2001). Considering the relatively higher amount of plasmalogens in the cell membranes, it is thus assumed that plasmalogens may potentially have significant effects on the physicochemical properties, and the integrity of the cell membranes.

## Role of Plasmalogens as an Internal Antioxidative Defense Agent in Biomembranes

Since their discovery, it was evident that, in contrast to ester-bonded phospholipids, vinyl-ether bond present in plasmalogens contribute to oxidation/antioxidant activity (Zoeller et al., 1999) as vinyl-ether bond preferentially decomposes during oxidation (Khaselev and Murphy, 1999). More specifically, it was proposed that plasmalogens could play a protective role against lipid peroxidation as a sacrificing/scavenger agent (Sindelar et al., 1999; Zoeller et al., 1999). In a study carried out by Sindelar et al. (1999), oxidative stress was applied to brain phospholipids with and without the presence of plasmalogens in separate liposomal systems. The results revealed that biomarkers for lipid peroxidation were significantly decreased in brain phospholipids with plasmalogens. Further, upon exposure to high oxidative conditions in normal cells, the plasmalogen levels were shown to decrease, suggesting a possible function as scavengers (Brosche and Platt, 1998; Brites et al., 2004).

Plasmalogens may protect biomembranes from environmental hazards as well. Hoefler et al. (1991), showed that plasmalogen deficient cells from patients with peroxisomal biogenesis disorders were significantly more sensitive to UV radiation exposure as compared to control cells. This observation supported by an *in vitro* study showed that plasmalogens-rich monolayer protects unsaturated lipids against UV-induced oxidation in the biomimetic system (Morandat et al., 2003). These observations imply that plasmalogens might protect polyunsaturated fatty acids of biomembranes from oxidative damage. Additionally, it has been reported that biomembranes relatively rich in plasmalogens exhibit low lipid peroxidation markers (Deng et al., 2017) and may provide protective shelter for biomolecules under oxidative stress conditions (Deng and Almsherqi, 2015).

The presence of vinyl-ether bonds in plasmalogens is probably responsible for providing protection against oxidative stress conditions. Indeed, vinyl ether bonds, the target of several oxidants including singlet oxygen, metal ions, and peroxyl radicals, are more susceptible to oxidation than ester bonds in phospholipids (Brites et al., 2004). However, peroxidation of vinyl-ether bonds by the free radicals might either be less efficient or more stable to abstract the hydrogen ion than alkyl radicals produced during the peroxidation of polyunsaturated fatty acids (Sindelar et al., 1999). It is also possible that during the process, the oxygenated vinyl ether radicals could be dissociated into water-soluble radical compounds leading to the inhibition of the chain reaction and stopping further propagation of lipid peroxidation (Sindelar et al., 1999). Therefore, plasmalogens that are present in sufficient concentrations in the cell membranes and able to cease lipid peroxidation could be classified as efficient internal antioxidants.

## Effects of Plasmalogens on the Physicochemical Properties of the Biomembranes

In addition to protecting phospholipids or lipoprotein and other components of the biomembranes against the damaging effects of reactive oxygen species, a substantial amount of research work supporting the proposition that alteration of the physicochemical and structural properties of the biomembranes could be influenced by the quantity and subtype of their plasmalogens content. *In vitro* studies carried out by Angelova et al. (2021) revealed that ethanolamine plasmalogen (C16:1p-22:5n6 pPE) promotes double-diamond cubic phase whereas primitive cubic phase and inverted hexagonal (H_*II*_) are observed in the DPA-ethanolamine plasmalogen (C16:1p-22:5n6 pPE) derivative. Complex morphological architectures such as cubic-lamellar liquid crystalline phases are established in DPA-plasmenyl phosphocholine (C16:1p-22:5n6 pPC)/MO (Monoolein) mixture, while ethanolamine plasmalogen (C16:1p-22:5n6 pPE)/DOPC (Dioleoylphosphocholine) bilayers promote a mix of multilamellar vesicular or whorls architectures (Angelova et al., 2021). The effect of cations on plasmalogen-rich membranes was studied by Cullis et al. (1986). Their experimental data showed that adding Ca^2+^ to a lipid bilayer consisting of negatively charged phospholipids and plasmalogens could alter and convert the flat membrane to a non-lamellar structure. Additionally, the authors have also observed that the addition of Ca^2+^ could promote membrane fusion in plasmalogen-rich membranes. Glaser and Gross (1994) reported that *in vitro*, vesicles with varying ratios of different plasmalogens could induce different non-lamellar structures. They have also demonstrated that different lipid compositions may affect the rate of membrane fusion, suggesting that plasmalogens are able to promote the formation of non-lamellar structures, which can further facilitate the membrane fusion process (Han and Gross, 1990, 1991; Glaser and Gross, 1994).

Lohner (1996) suggested that the tendency of plasmalogens to form highly curved structures such as inverted hexagonal may be attributed to their physicochemical properties. The lack of the carbonyl oxygen in position sn-1 of ether lipids affects the hydrophilicity of the headgroup and allows stronger intermolecular hydrogen bonding between the headgroups of the lipids which promotes the formation of non-lamellar structures. This suggestion is supported by studies that show that model membranes composed of plasmanyl and plasmenyl ethanolamines exhibit lower gel to liquid phase transition compared with diacyl glycerophosphoethanolamine analog (Paltauf, 1994). Detailed studies demonstrate that the optimum transition temperature required for the transformation from lamellar to hexagonal phase is lowered in the presence of plasmalogen (Lohner et al., 1991). Further experimental work showed that plasmalogens are an essential component in regulating several membranous activities (Hermetter et al., 1989; Glaser and Gross, 1995; Braverman and Moser, 2012) such as membrane fusion processes (Brites et al., 2004). Furthermore, physiological studies have shown that the highly heterogeneous bilayers enriched in plasmalogens present in synaptic vesicles (Breckenridge et al., 1973; Braverman and Moser, 2012; Dorninger et al., 2019) support neurotransmitter release and vesicular fusion is very sensitive to the amount and type of ethanolamine plasmalogens (pPE) content. Any minor reduction in either the vinyl-ether and/or the polyunsaturated fatty acid content of vesicles would result in a significant reduction in the number of successful membrane fusion events (Rog and Koivuniemi, 2016). Thus, this mechanism alone might be adequate to rationalize the correlation between the decreased membrane pPE and the neuronal function decline associated with neurodegenerative diseases and aging.

## Plasmalogen Distribution in Biological Membranes

Effects of plasmalogens on artificial membrane architectures have motivated further studies on their molecular distribution and packing in simulations of lipid membranes comprising plasmalogens and palmitoyl-oleoyl-phosphatidylcholine. Ether lipids in general, and plasmalogens in particular, form more tightly packed membranes than their diacyl analog. Specifically, for pPE, this effect could be attributed to the highly ordered state of sn1 chain and closer packing of the sn1 and sn2 chains where the orientation of the alkyl chain and vinyl-ether linkage of plasmalogens dictate the increased packing of membranes (Han and Gross, 1990; Rog and Koivuniemi, 2016). As such, it is expected that the bilayer thickness, lateral phase segregation, rigidity, and other physicochemical properties of the biomembranes are likely to be influenced by the amount and distribution of plasmalogens (Rog and Koivuniemi, 2016).

Experimental work using human red blood cells showed that the labeled pPE spontaneously spread from the outer to the inner leaflet with a transition half-time comparable to the corresponding diacyl analog. At equilibrium, about 80% of both subclasses of PE were in the inner leaflet whereas only 20% of choline plasmalogen (pPC) was translocated to the inner leaflet (Fellmann et al., 1993). It was further reported that in the sarcolemmal membrane, ethanolamine and pPC were asymmetrically distributed, with the plasmalogens predominating in the inner leaflet (for example, with pPE accounting for 44% and 24% of ethanolamine glycerophospholipids in the inner leaflet and the outer leaflet, respectively; Kirschner and Ganser, 1982; Post et al., 1988). Plasmalogens and diacyl glycerophospholipids segregation can occur laterally as well but this has not been reported in artificial membrane models. If it happens in biological membranes, then it is most likely to be caused by the specific interaction with membrane proteins. It has been speculated that asymmetrical distribution of pPE at the sarcolemmal membrane might be essential for Ca transport (Bick et al., 1991) and *trans*-sarcolemmal sodium-calcium exchange (Ford and Hale, 1996). Interestingly, plasmalogens localized in the inner layer of the plasma membrane are critical for sensing the cellular level of plasmalogens and they play an important role in the regulation of plasmalogen biosynthesis (Honsho et al., 2017). Plasmalogen levels in the cell membranes could affect the intracellular cholesterol distribution (Rodemer et al., 2003) and transportation within biomembranes (Munn et al., 2003).

## Effects of Plasmalogens on Alteration of Biomembrane Morphology

Notwithstanding the significant advances achieved in understanding the role of plasmalogens in the artificial membrane organization (Feller, 2008; Gawrisch and Soubias, 2008; Wassall and Stillwell, 2008), similar studies on the effects of plasmalogens on biomembrane curvature and architecture *in vivo* are limited mainly because of the deficiency of suitable experimental techniques to match the membrane geomorphologies in the cell with specific local membrane lipid composition.

One of the earliest investigations relating plasmalogen to membrane morphology alteration was reported by Teichman et al. (1972) who discovered that mammalian spermatozoa retained highly osmiophilic material when fixed with glutaraldehyde containing malachite green or pyronine. Further analysis of malachite-green-affinity (MGA) material found that MGA consists largely of pPC (Teichman et al., 1974). Although the highly condensed, plasmalogen-rich MGA material has various morphologies, its homogenous and apparently membranous whorls resembling myelin sheet was the most common architecture observed.

**FIGURE 2:**
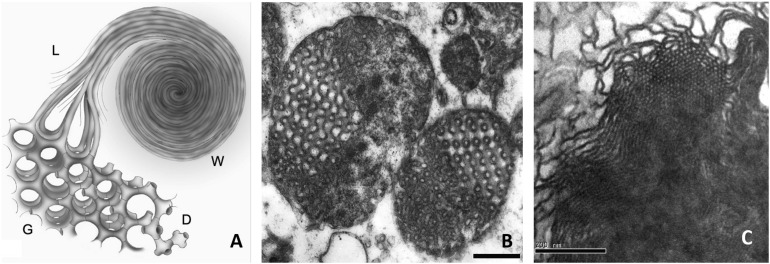
**(A)** Schematic diagram depicting a model of continuous membrane folding represents the formation of double diamond (D) and gyroid (G) cubic type, hexagonal and lamellar structures (L), and whorls (W) membrane organizations. Cubic membrane organizations have been reported to be rich in plasmalogens. **(B)** Transmission electron microscopy images of cubic membranes transformation of the mitochondria in amoeba *Chaos* cells under starvation stress condition and **(C)** Liposomes generated from the lipids extracted from amoeba *Chaos*, typically display cubic and hexagonal phase organization. **(A,C)** reprinted from Almsherqi et al. (2009) with permission.

Deng et al. (2009) have demonstrated that during cell starvation, the significant increase in pPC in amoeba *Chaos* was associated with the highly organized and curved cubic membrane formation (Figure 2). An unusually high concentration of PUFA has been found in lipid analysis of amoeba *Chaos* (C22:5; DPA; Deng et al., 2009). Three main lipid species, namely pPE (C16:0p/C22:5), pPC (C16:0p/C22:5), and diacyl-PI (C22:5/C22:5), were detected in amoeba *Chaos* lipid extracts. Interestingly, the liposomal constructs obtained from the amoeba *Chaos* lipid extracts, typically display cubic and hexagonal phase organization (Figure 2).

Upon cubic membrane formation, under starvation stressed conditions, the relative amounts of PUFA especially DPA increased up to 1.6-fold (Almsherqi et al., 2010), at the expense of linoleic acid (all-*cis*-9,12-octadecadienoic acid; C18:2 omega-6). This suggests a metabolic link via the omega-6 DPA pathway giving rise to omega-6 DPA, rather than omega-3 DPA, which requires Δ15 desaturation of the acyl chain that is absent in animals (Deng et al., 2009). Furthermore, it has been suggested that cubic membranes which harbor a significant amount of plasmalogens (Deng et al., 2009), might act as a “defensive” shield to mitigate the oxidation of biologically vital macromolecules (e.g., lipids, oligonucleotides, and mRNAs; Almsherqi et al., 2008; Deng and Almsherqi, 2015; Deng et al., 2017). In this light, further studies carried out on amoeba *Chaos* showed that when oxidation was imminent in the mitochondria (Wang et al., 2011; Rambold et al., 2015) during cell starvation, the percentage of cell survival in amoeba *Chaos* with cubic membrane (rich in plasmalogens) was significantly higher than those amoeba without mitochondrial cubic membrane transformation (Chong et al., 2018). This suggested that plasmalogens play a role not only in membrane transformation but also as a cytoprotectant to promote cell survival. Similar results were reported in *C. elegans* where the lifespan was reduced by 30% in three different point-mutant strains that resulted in a near-complete loss of plasmalogens. Furthermore, the ability to survive and grow in cold ambient temperature was significantly reduced from 60% in the wild type *C. elegans* to 5–20% in the mutant strains.

## Changes in Cellular Membrane Morphology Arising From Exogenous Metabolic Precursors and Plasmalogens Supplements

Based on clinical, experimental, and biomimetic simulation observations, alterations of plasmalogen levels and membrane transformation are associated. Thus, the availability of plasmalogens (as a result of supply/deficiency) could influence biomembrane transformation, during cellular/organelle stress conditions.

Experimental data demonstrated that alkylglycerols supplementation to rodents was utilized for ether lipids synthesis including plasmalogens (Hayashi and Oohashi, 1995). Furthermore, adding alkylglycerols to the culture media of plasmalogen-deficient CHRS, RCDP, or CHO cells reinstated the normal plasmalogen levels (Liu et al., 2005). Therefore, it can be deduced that dietary precursors such as alkylglycerols can be utilized for plasmalogen synthesis and restoration of normal levels of ether lipids in various tissues.

**FIGURE 3:**
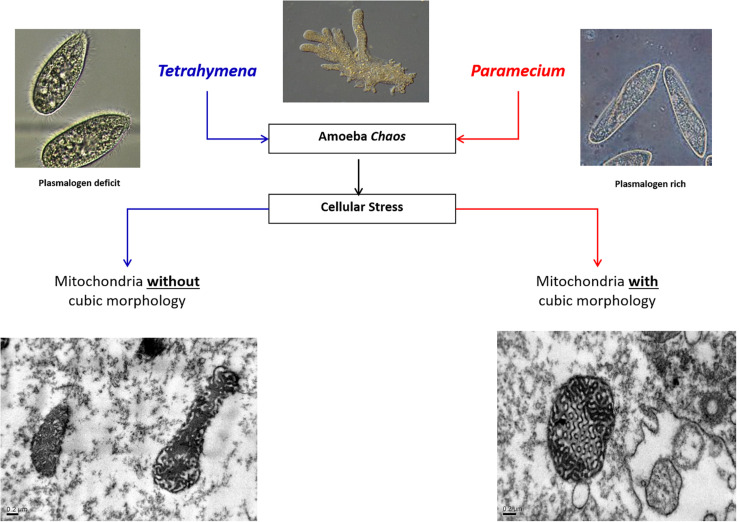
Schematic diagram depicting the effect of food supplements on the mitochondria membrane morphological transformation in amoeba *Chaos*.

Plasmalogen dietary supplementation may aid membrane morphology alteration, particularly in amoeba *Chaos*. Lipid profile data of amoeba *Chaos* food organisms revealed significant differences in the plasmalogen levels of the two food organisms, namely Paramecium multimicronucelatum (hereinafter referred to as Paramecium) and Tetrahymena pyriformis (hereinafter referred to as Tetra). In particular, pPC was in relatively high abundance in Paramecium compared to Tetrahymena. The higher level of pPC in amoeba *Chaos* cells was observed in Paramecium-fed but not Tetra-fed cells (Deng et al., 2009). Interestingly, feeding amoeba *Chaos* with the extracted lipids from Paramecium (pPC-rich) or polyunsaturated fatty acids, specifically omega-6 DPA, (Deng et al., 2009) were able to induce mitochondrial inner membrane rearrangements into cubic morphology (Figure 3). Furthermore, liposome construct using the extracted lipids from amoeba *Chao*s exclusively fed with Paramecium do induce cubic or hexagonal organization *in vitro* (Deng et al., 2009; Figure 3).

In a parallel study adopting a similar technique, MO/DOPC nanostructured lipid phases with alteration of plasmalogens carrying DPA (C22:5n-6) were used in order to validate the potency of plasmalogens on the membrane curvature and/or membrane re-arrangements (Angelova et al., 2021). The results showed that plasmalogens-carrying C22:5n-6 fatty acids at sn-2 position effectively curved the lamellar phase structures and induced multiple nanostructures such as inverted hexagonal (H_*II*_), double diamond cubic phase, double-membrane vesicles, and multilamellar whorl topologies indicating the importance of DPA-based PE and PC plasmalogens in inducing membrane curvature (Angelova et al., 2021). These observations suggest that plasmalogens and PUFAs (C22:5n-6) each play a role in lipidic phase transition *in vitro* and cubic membrane transformation *in vivo*. Plasmalogens might work synergistically in transitioning the lipid phase to higher-order morphologies. With relevance to biological membrane transformation, it is highly possible that *in vivo*, lipids, proteins, and other ionic milieu or pH factors partake in a morphological transformation of biomembranes.

Translational studies of plasmalogens supplementation in Alzheimer’s disease revealed that there were improvements in the learning ability and cognitive function of the rodents when fed with plasmalogen-rich lipids (Yamashita et al., 2017) and significant improvement was observed in mild/early-stage Alzheimer’s patients when treated with oral supplements of scallop-derived plasmalogens (Fujino et al., 2017, 2018). However, the bioavailability of oral supplements of plasmalogens or plasmalogen precursors to the neuronal cells is still under investigation. Plasmalogens are carried in the plasma via chaperone proteins like LDL (Wiesner et al., 2009) and delivered to the tissues through LDL receptor-mediated pathway (Dehouck et al., 1997; Candela et al., 2008). Although some studies suggest that plasmalogen precursors (Wood et al., 2011), and DHA in the form of phospholipids can pass through the blood-brain barrier (BBB; Lagarde et al., 2015), plasmalogens may not be able to cross the BBB efficiently. These studies indicate that the brain tissue relies on the *de novo* synthesis of ether lipid rather than on the transportation of peripherally synthesized plasmalogens across the BBB. However, depleted levels of brain DHA, and presumably DHA-containing plasmalogens, could be replaced by sustained DHA supplementation (Salem et al., 2001). Enriching neuronal cell membranes with plasmalogens could improve neuronal function through modulation of non-lamellar membrane transformations and synaptic plasticity (Paul et al., 2019).

## Experimental Models to Investigate Plasmalogens Effect on Biomembranes

Understanding the role of plasmalogens in biological membranes is key to understanding how plasmalogen depletion may contribute to the onset and progression of pathological conditions. Several knockout (KO) mice models were generated for *in vivo* studies, namely, Pex7 KO mouse (Brites et al., 2011), Pex7 hypomorphic mouse (Braverman et al., 2010), Gnpat KO (Dorninger et al., 2019), Agps hypomorphic mouse (Liegel et al., 2014), and recently, PEDS1 KO mice which lack both plasmanylethanolamine desaturase activity (the key enzyme involved in the initial step of plasmalogen synthesis) and plasmalogens (Werner et al., 2020). A sole mutation of an enzyme leading to a specific plasmalogen deficiency offered by the experimental models could be advantageous for a clear-cut understanding of the biochemistry-pathology relationship.

In addition to KO mice models and human neuronal cell culture, a nematode, *C. elegans*, has lately been established as a model for plasmalogen deficiency (Drechsler et al., 2016; Shi et al., 2016). Unlike murine KO mutants, which are often infertile, the ether lipid mutants *C elegans* are viable and fertile, which offers a valuable model to study severe forms of plasmalogen deficiencies. *C. elegans* is also a suitable model to incorporate dietary tracers which could be detected in the cell membranes and allow detailed measurements of ether lipids distribution in the cell membranes (Dancy et al., 2015).

Free-living giant amoeba *Chaos* could also be proposed as an experimental cell model system to assess the effect of plasmalogen deficiencies on cellular membrane structure and function. In addition to the high cellular dynamic (exocytosis, endocytosis, and pinocytosis), the advantage of studying membranes transformations in amoeba *Chaos* is the ability to trace membrane morphological changes in relation to changes in the diet. Modifying the quantity/quality of the food supply is feasible and controllable (Tan et al., 2005). Further studies could establish a relationship between the exogenous plasmalogens supplementation and cellular membrane activity.

## Significance of Studying the Effect of Plasmalogen on Membrane Morphology

Identification of a mechanistic association between the reduction in plasmalogens within cellular membranes and deterioration of vital cellular functions is important in two aspects: early detection of cellular dysfunction before it progresses to the disease state and secondly, proper planning for prevention and/or therapeutic intervention.

First, plasma plasmalogen levels could be used as a biomarker to indicate the onset of cellular dysfunction and monitor the rate and extent of the deterioration or improvement (Fernandes et al., 2020). Detecting plasmalogens in the blood, for example, would be an advantage over other methods of detection (Fernandes et al., 2020). Second, restoring plasmalogen levels (by oral supplements for example) at the appropriate intervention time could potentially delay or prevent the onset of cellular dysfunction that is associated with neurodegenerative and/or age-related diseases. Restoration of plasmalogens for specific tissues requires an improvement in the bioavailability of plasmalogens or plasmalogen precursors, enhanced BBB penetration ability (e.g., using liposome-based strategies; Vieira and Gamarra, 2016), and the selection of a proper stage (preclinical, early/late clinical) to start the treatment.

## Concluding Remarks

In conclusion, with the potential role of plasmalogens in biomembranes as a modulator of cell membrane physicochemical properties and morphology, plasmalogens cannot be regarded to function only as an internal antioxidant that maintains membrane lipids and proteins integrity. Plasmalogens affect both across the lipid bilayer (lipid asymmetry) and lateral dimension (lipid domains) and facilitate the formation of non-lamellar structures in the cell. Inducing membrane curvature initiates and promotes membrane fusion/fission, vesicular formation, and molecular transportation which are crucial for normal cell function (especially neuronal cells) and adaptation to stress conditions. This new aspect of plasmalogens in the bilayer membrane architecture has been tested in artificial membrane lipid systems and yet to be explored *in vivo*. Furthermore, in-depth study of the underlying molecular mechanism of inducing and selecting a specific spatial arrangement of bilayer-based membranes is indeed critical to the understanding of the association between cell membrane alteration and adjustment of cellular function and adaptation to stress and pathological conditions.

## Author Contributions

Part of the experimental work cited in the manuscript has been done in the author’s laboratory. ZA contributed to the manuscript writing and preparation of figures.

## Conflict of Interest

The author declares that the research was conducted in the absence of any commercial or financial relationships that could be construed as a potential conflict of interest. The handling editor declared a shared membership in a society with the author at time of review.
